# Use of Precision Feeding during Lactation Improves the Productive Yields of Sows and Their Piglets under Commercial Farm Conditions

**DOI:** 10.3390/ani14192863

**Published:** 2024-10-04

**Authors:** María Aparicio, Natalia Yeste-Vizcaíno, Joaquín Morales, Nerea Soria, Beatriz Isabel, Carlos Piñeiro, Antonio González-Bulnes

**Affiliations:** 1 Animal Data Analytics, S.L., C/ Dámaso Alonso 14, 40006 Segovia, Spain; joaquin.morales@ada-animaldata.com (J.M.); carlos.pineiro@ada-animaldata.com (C.P.); 2 Cuarte S.L., Grupo Jorge, Ctra. de Logroño km 9.2, Monzalbarba, 50120 Zaragoza, Spain; nereasoria@cuartesa.com (N.S.); antonio.gonzalezbulnes@uchceu.es (A.G.-B.); 3 Faculty of Veterinary Medicine, Universidad Autónoma de Barcelona, UAB, Cerdanyola del Vallés, 08193 Barcelona, Spain; 4 Faculty of Veterinary Medicine, Universidad Complutense de Madrid, UCM, Ciudad Universitaria s/n, 28040 Madrid, Spain; bisabelr@ucm.es; 5 Faculty of Veterinary Medicine, Universidad Cardenal Herrera-CEU, CEU Universities, C/Tirant lo Blanc, 7 Alfara del Patriarca, 46115 Valencia, Spain

**Keywords:** lactation, nutrition, precision feeding, sustainability, swine

## Abstract

**Simple Summary:**

This study reports the results of two studies aiming to determine, under commercial farm conditions, the effects of electronic sow feeders on the production and economic yields of lactating sows. The results indicate remarkable technical and economic outputs compared with traditional feeders due to the weaning of heavier piglets with a lower amount of feed per kg of weaned piglet.

**Abstract:**

Adequate nutritional management in maternities is one of the most challenging aspects of swine production. This study reports the results of two studies aiming to determine, under commercial farm conditions, the effects of precision feeding (electronic sow feeders, ESFs) on the production and economic yields of lactating sows and possible nutritional and metabolic differences when compared to a control group fed with traditional feeders. The first trial showed that sows fed with ESFs weaned heavier piglets than sows fed with traditional feeders. Feed intake during the lactation period was similar in the sows of both groups; consequently, the amount of feed per kg of weaned piglet was lower in the sows fed with ESFs, which is a remarkable economic output. The second trial confirmed these findings and showed that, despite similar feed intakes, the sows fed with ESFs had lower bodyweight losses during the lactation period, but there were no major differences in milk composition or metabolic traits of sows and piglets.

## 1. Introduction

Sustainability is currently one of the main keywords in swine production. In the current situation, the original concept of environmental sustainability has been reinforced by the concepts of social and economic sustainability (profitability). Hence, producers are dealing with new challenges and developing new strategies to improve sustainability without prejudicing efficiency by reducing costs and impact while increasing benefits.

One of the main factors affecting efficiency is numerical productivity (average number and weight of piglets weaned per productive sow and year), which directly affects production costs and profitability [1]. Improvement of production efficiency has been afforded by increasing the number of piglets per sow and year by both diminishing non-productive periods and increasing litter size. An increase in litter size has been afforded by the selection of hyperprolific strains with large litters. Consequently, modern sows commonly wean 33–35 piglets per year, and some herds may even wean more than 40 piglets per year per sow [2]. However, hyperprolificacy was early related to the appearance of low-birth-weight (LBW) offspring and, consequently, decreases in postnatal survival and development of the offspring. LBW offspring have a subsequent impact on productivity because of their predisposition to disease and reduced growth potential, with a longer period for achieving market weight than their littermates and poor meat quality [3,4,5].

A concomitant factor with a major influence on profitability is nutrition, through both feed cost (which accounts for up to 75% of total swine production cost [6]) and effects on pig performance. Specifically, maternal nutrition and feed intake during the suckling period may support adequate growth of piglets reared in large litters during this nursing phase [7]. Careful management of sow feeding during lactation can markedly optimize feed intake, facilitating greater milk yields and maximizing the number and weight of weaned piglets, including LBW piglets, for improving output and profitability at the farm level [8].

These factors affect farm profitability and economic sustainability, making clear the strong need for useful strategies. Precision livestock farming (PLF) has emerged as one of the most representative improvements. The objective of PLF is to provide farmers with tools for online and continuous monitoring of the status of the animals and their environment [9], allowing for a continuous and easy assessment of the health, behavior and welfare of the animals [10,11].

The adequate management of maternities and hyperprolific sows, mainly the feeding strategy, is one of the most challenging points in swine production. Feed intake and feed intake behavior are arising as critical aspects of the productive efficiency during lactation due to the metabolic needs of the sow. The metabolic demand of lactation is one of the most critical points for breeding sows because it may even triple the metabolic demand of pregnancy [12,13]. Farmers and nutritionists are responsible for addressing nutritional requirements and the proper management of sows during different periods of their reproductive cycle [14]. However, at times, sows may be either underfed, inducing body reserve mobilization, or overfed, increasing nutrient excretion [15]. In this scenario, precision feeding (PF) could help to simplify the adaptation of adequate feeding strategies according to the body condition of the sows. PF is a technique that improves the utilization of feed and nutrients and thus reduces feeding costs, nutrient excretion and environmental impacts by providing an adequate amount of nutrients at the right time to each animal [16,17]. PF is based on nutritional models used to predict nutrient requirements, sensors used to control animal performance and electronic sow feeders (ESFs) so that the quantity and quality of the diet can be individualized [15].

Thus, efficient systems to manage feed intake during lactation have been the focus of intense research [13,18,19]. It is currently widely accepted that the best option to feed lactating sows is to establish a basic or prefixed curve which, managed by the farmer, would avoid sudden drops or excess of feed intake. The best way to implement this curve would be ad libitum access to feed using modern systems, such as the ESF tested in our trials, which allows both the sows and the farmer to individually adjust the basic curve and the ration to the feed intake of the sow, with the final aim of providing the adequate feed amount for controlling and minimizing body tissue mobilization but also for avoiding feed excess and hence indigestion causing sudden drops in intake and metabolic disruptions [20].

In this way, ESFs are good tools for incorporating PF into farm strategies and improve economic and environmental sustainability by minimizing the impact of pig production through improving the efficiency of feed and nutrient use. PF also improves economic sustainability by increasing profitability through reducing feed costs and increasing the performance of animals. PF may also improve social sustainability by increasing the use of technology of farms, which may be more attractive to younger farming staff and may increase the equality of opportunities and social inclusion.

However, there are significant factors to be mastered for translating research into farm strategies and, consequently, for guaranteeing the best balance between advantages and investments. Hence, this study reports the results of two successive studies. The first trial aimed to determine, under commercial farm conditions, the effects of PF on productive and economic yields of lactating sows. The second trial aimed to determine possible events related to maternal metabolic traits, milk quality and postnatal development and metabolism of the offspring which may be involved in the improvements in the productive yields obtained in the first trial.

## 2. Materials and Methods

### 2.1. First Trial: Effects on Productive and Economic Yields of Lactating Sows

The first trial was designed as a multicenter study, which was carried out from June to November 2023 on six commercial farms located in Central Spain. No ethical approval of the animal trial was necessary since only data on sow intake and piglet weight were obtained. A total of 368 sows (Large White × Landrace; between 36 and 112 sows per farm), from parity 1 to 7 and inseminated by Piétrain boars, were used.

The management of sows and their offspring followed standard commercial farm practices, which involved housing them indoors under controlled temperatures. Sows in all the farms were classified according to their parity, condition score and historical productive data to ensure that females with similar features were used in each treatment. The treatments were established from one week before delivery to the day of weaning, when half of the sows were restrained in farrowing stalls equipped with electronic sow feeders giving six rations per day (Group ESF; Gestal SOLO, Jyga Technologies Inc., Quebec, Canada; n = 178; feed given at 5 AM, 8 AM, 11 AM, 14 PM, 17 PM and 20 PM), while the remaining sows were restrained in farrowing stalls equipped with traditional feeders (sow bowls) providing feed in two daily rations (Group Control or CON; SB, Rotecna, Agramunt, Spain; n = 190; feed given at 8 AM and 14 PM). However, the final portion, the bowl feeder, was the same in all the crates for both groups.

All sows, independently of the farm and experimental group, were fed with standard soy grain-based diets [21] (Supplementary Table S1). The sows in both the CON and ESF groups followed a reference basic feed intake curve, increasing the amount of feed offered to the sow from the day of farrowing until day 11 post-farrowing and establishing afterwards a plateau until the day of weaning, after 24 days of lactation. However, the amount of feed served in Group CON could be adjusted manually by the staff of the farm depending on the previous day’s feed intake, while the amount of feed in Group ESF was automatically adjusted using the device, giving the sow the opportunity to eat 20% more feed each time. Feed intake and refusal by sows were also individually and daily recorded (manually in the case of Group CON and automatically in the case of Group ESF) from the day of farrowing to that of weaning; therefore, the total feed intake during the lactation period and the amount of feed per kg of weaned piglet were determined.

Productivity data were recorded per sow within 24 h after birth including the total number of live and stillborn piglets, and weight scales were used for recording both the weights of individual piglets and the total litter weight. A total of 4852 live piglets were involved in this study. Productivity data, including the total number of weaned piglets (2063 in Group ESF and 1996 in Group CON) and individual and litter weights, were recorded again at weaning. Average Daily Weight Gain (ADWG) was individually determined for the total lactation period, using the formula ([final weight−initial weight]/number of days).

### 2.2. Second Trial: Effects on Metabolic Traits and Milk Quality of Sows and Growth and Metabolic Traits of Piglets

The second trial was designed, after the results were obtained in the first study, to understand the possible effects of PF on the metabolic traits and milk quality of sows, and the growth and metabolic traits of their piglets. Consequently, an experimental procedure was carried out on a commercial farm (San Pedro farm, Plasencia de Jalón, Zaragoza, Spain), according to the European Union Directive and the Spanish Policy for Animal Protection RD53/2013. The Committee of Ethics in Animal Research of Universidad Complutense de Madrid (UCM) approved the experiment (CEEAH2788M2). A total of 12 sows (Danbred), from parity 2 to 7 and inseminated by Piétrain boars, were used.

Six sows were individually restrained in farrowing stalls equipped with electronic sow feeders (Group ESF; parities: 3, 3, 4, 5, 5, 6), while six sows were housed in crates equipped with traditional feeders (Group CON; parities: 3, 3, 4, 5, 5, 6) from the week before parturition to the day of weaning (four weeks after delivery); again, the final portion, the bowl feeder, was the same in all the crates for both groups. Sows were distributed across the treatment groups (either ESF or CON) considering their parity but also their condition score and historical productivity data to ensure that females with similar features were used in each treatment. Sows in both groups, similarly to the first trial, were fed with standard soy grain-based diets (Supplementary Table S1) following the basic feed intake curve and timetable previously described, adjusted manually depending on the previous day’s feed intake in Group CON and automatically in Group ESF.

At birth, productivity data were recorded per sow, including total numbers of live and stillborn piglets and the total weight of the litter using a litter scale (MPigData, Mensoft, Madrid, Spain). A total of 197 live piglets were sexed and weighed to be involved in the trial after performing individual identification with eartags, within-group cross-fostering and allocation to mothers at a rate of around 16 piglets/sow. Piglets were weighed again at weaning, after 24 days of lactation. Average Daily Weight Gain (ADWG) was individually determined for the total lactation period, using the formula ([final weight—initial weight]/number of days).

The weight and body condition (back-fat depth) of all the sows were measured at the beginning (i.e., one week before parturition) and at the end of this study (i.e., day of weaning, around four weeks after delivery). Back-fat depth was measured at the P2 point, which lies on the right side of the animal at 4 cm from the midline and transversal to the head of the last rib, using ultrasound equipment fitted to a multifrequency linear array probe (ProVetScan SF2 Wireless scanner, NewVetec, León, Spain). The initial bodyweight and final bodyweight of the sows were used to calculate weight losses during lactation. The resulting values have the limitation that bodyweight prior to delivery includes the weight of fetuses, amniotic fluids and afterbirth, but we were constrained by welfare issues and management procedures in the farm so we could not move the animals immediately after delivery. Nonetheless, the bodyweight of the sows and the litter size were similar between groups, so we can assume a similar deviation in both groups.

Samples of milk were taken at 14 days after farrowing to obtain representative data at mid-lactation. In brief, at mid-morning, the milk was collected from all functional glands by hand-milking after the piglets were separated from sows and allowed afterwards to join the mother for stimulating milk letdown, and sows were injected with 20 I.U. oxytocin (Oxitocina Diana, Super’s Diana S.L, Barcelona, Spain). Immediately, 25 mL of milk was stored in 50 mL falcon tubes at −80 °C until assayed for lactose, protein and fat content, using an IR spectrophotometer (MilkoScan ™ 7RM, Foss Iberia, Barcelona, Spain).

On the same day, day 14 of lactation, when creep feeding was allowed to the piglets, blood samples were obtained from 96 piglets (48 piglets in each group; 4 females and 4 males in each litter), representative of the mean size of the litter. These same piglets were sampled again at weaning, when blood samples from their mothers were also drawn. Blood samples were drawn by puncture of the cranial vena cava (*cava cranialis*) using EDTA vacuum tubes (Vacutainer Systems Europe, Meylan-Cedex, France) and immediately centrifuged at 1500× *g* for 15 min (Nahita-Blue, Innovagen, Madrid, Spain). Plasma was immediately separated and stored at −80 °C until required for analyses of glucose and lipid metabolism. Plasma concentrations of glucose (glucose and fructosamine), protein (total protein content) and lipids (total cholesterol, high- and low-density lipoprotein cholesterol (HDL-c and LDL-c, respectively) and triglycerides) were assessed with a clinical analyzer (Konelab 20i, Thermo Fisher Scientific, Madrid, Spain).

### 2.3. Statistical Analysis

The analyses assessed the effects of the diet on the weight and metabolic condition of sows and the developmental traits and metabolic status of their piglets. The sows and piglets were considered the experimental units. In the first trial, the analysis assessed effects from diet (CON vs. ESF) plus effects of farm, parity and bodyweight and condition at farrowing. In the second trial, the analysis assessed effects from diet plus effects of parity and bodyweight and condition at farrowing. Data were analyzed using SAS (Version 9.4; SAS Inst. Inc., Cary, NC, USA). Verification of normal distribution was completed using a Kolmogorov–Smirnov test (PROC UNIVARIATE), and afterwards, data were analyzed using an ANOVA test (PROC GLM) with Tukey adjustment. Changes over time were assessed using ANOVA for repeated measures with the Greenhouse–Geisser correction. Variables that did not fit a normal distribution were analyzed using a non-parametric Kruskal–Wallis test (PROC NPAR1WAY). All data are presented in this article as mean ± SEM. Statistical significance was accepted from *p* < 0.05, whereas a trend was considered when 0.1 > *p* > 0.05.

## 3. Results

### 3.1. First Trial: Effects of ESF on Productive and Economic Yields of Lactating Sows

#### 3.1.1. Productive Data at Birth

The overall analysis of all farms showed no significant differences in the numbers of total, live and stillborn piglets between sows in Groups ESF and CON. Moreover, there were no significant differences in the mean birth weights for the individual piglets and the total litter between Groups ESF and CON, as depicted in Table 1.

#### 3.1.2. Piglet Performance during the Suckling Period

At weaning, four weeks after delivery, the mean bodyweights for the individual piglets (*p* = 0.007) and the total litter (*p* = 0.030) were higher in the ESF than in the CON group (Table 2). The ADWG was therefore higher in the piglets of the ESF group, being 220.1 ± 2.86 g/day for ESF and 211.6 ± 3.06 g/day for CON (*p* = 0.040). The number of weaned piglets per litter was similar between groups.

#### 3.1.3. Sow Feed Intake and Costs during Lactation

As shown in Table 3, there were no differences in either the average daily feed intake or the total feed intake per sow during the lactation period between sows in Groups ESF and CON. Due to the heavier litters previously described, the amount of feed per kg of weaned piglet was significantly lower in Group ESF than in Group CON (*p* = 0.002).

### 3.2. Second Trial: Effects of ESF on Metabolic Traits and Milk Quality of Sows and Growth and Metabolic Traits of Piglets

#### 3.2.1. Productive Data at Birth

There were no significant differences in the number of total, live and stillborn piglets between sows in Groups ESF and CON, as depicted in Table 4. Moreover, there were no differences in the mean weights for the individual piglets and the total litter between the two groups.

#### 3.2.2. Maternal Performance

Mean bodyweight and back-fat depth were similar in sows allocated to the ESF and CON groups at the beginning of the experimental trial, one week before parturition (Table 5). The decrease in bodyweight during lactation induced a negative and similar ADWG in both groups, but a trend was found that sows in the ESF group had a higher bodyweight at weaning (*p* = 0.060).

#### 3.2.3. Metabolic Traits and Milk Quality

The analysis of milk quality (Table 6) and mean plasma concentrations for the indexes of glucose, lipids and protein metabolism in both sows (Table 7) and piglets (Table 8) showed similar values between groups. However, we have to highlight significantly higher values of HDL cholesterol and proteins in the piglets of Group CON at 14 days of lactation, which were not found afterwards.

#### 3.2.4. Piglet Performance during Lactation

A similar number of weaned piglets were found in both groups with no statistical differences, but the use of ESF was related to a higher ADWG in the piglets (*p* < 0.001), which reached a higher weaning weight than their counterparts in Group CON (*p* < 0.001). Consequently, as shown in Table 9, the total litter weight at weaning showed a tendency to be higher, by about 13.5 kg (+19.0%), in Group ESF than in Group CON (*p* = 0.090).

## 4. Discussion

The results of this study indicate that, under commercial farm conditions, the use of ESFs during lactation has a positive impact on the performance of the sows and their litters. The data from the first trial showed that when suckling a similar number of piglets with similar weights at the start of the lactation period, sows fed by ESFs weaned heavier piglets and therefore heavier litters than sows fed with traditional feeders. Feed intake during the lactation period was similar in the sows of both groups; consequently, the amount of feed per kg of weaned piglet was lower in the sows fed with ESFs, which is a remarkable economic output. The results of the second trial confirmed the higher ADWG and weaning weights of the piglets from sows fed with ESFs and indicate that, despite similar feed intakes, sows with ESFs showed lower bodyweight losses during the lactation period. On the other hand, the milk composition and the metabolic traits of sows and piglets were similar between groups, excepting lower—but within physiological values—plasma concentrations of HDL cholesterol and proteins in Group ESF at 14 days of lactation, not found again at weaning.

Group ESF showed higher ADWG during the suckling period (*p* = 0.04 and 0.0008 for the first and second trials, respectively) and therefore higher weaning weight (*p* = 0.007 and 0.0006 for the first and second trials, respectively), which should be related to a higher intake of nutrients, due to either higher production or better quality of the milk. Previous studies have indicated that milk yield is the main factor affecting the growth of suckling piglets [22], with a strong positive correlation between amount of milk produced and growth of piglets. Milk production depends primarily on the sow’s genetic potential and its feed intake, but it is strongly stimulated by litter size and the suckling capacity of piglets [23]. Milk production was not assessed in the current trial, and it should certainly be addressed in future trials, but we assessed possible differences in the quality of the milk between sows in Groups ESF and CON in the second trial. The results showed no significant differences in the main components of the milk, although the concentration of lactose was higher in Group ESF, without reaching statistical significance, and the concentrations of protein and fat were lower. These results should be taken only as data paving the way for further study with more animals and samples but, indirectly, these data suggest a higher production of milk in ESF sows since, across species, the concentration of lactose in milk has a strong positive correlation with overall milk volume [24], while the percentage of fat is negatively related to the amount of milk produced [25]. Hence, although this finding needs further research, we can hypothesize a higher volume of milk produced by sows in Group ESF as the main cause for the higher ADWG found in their piglets. Interestingly, one of the possible reasons for higher milk production in sows fed with ESFs may be related to the behavior of the lactating females. Krogh et al. [26] reported that the amount of milk produced by a sow is related to the amount of time that it spends standing, due to a diminished mammary blood flow when sows are lying. Hence, in the case of retrained sows as in this study, sows with ESFs or sows that are stimulated to move and stand by the farmers produce more milk than sows lying down, as in the sows of Group CON, which only stood to drink or to eat when the feed was served. On the other hand, the quality of milk is also affected by intake behavior [27]. The work of Nielsen et al. showed a higher percentage of lactose in sows fed three times per day with ESF systems at a slow speed than in traditional feeders allowing three meals but at a high speed [27].

Intake behavior also affects feed consumption, which is composed of feed intake and feed wastage. In commercial farms, it is well known that food wastage is one of the main critical problems for economic efficiency, and ESFs are seen as a possible solution. In our trials, the total amounts of offered feed were the same for the CON and ESF groups but ESF sows may waste less feed than CON sows (there is a smaller amount of food each time, so there are fewer opportunities for wastage). In agreement with this finding, in the first trial of this study, the feed consumption in Group CON was numerically higher than in Group ESF, and, consequently, the feed consumed per kg of weaned piglet was significantly higher than in the ESF group (*p* = 0.002). A higher maternal consumption of feed with a lower weaning weight of the piglets, as found in the study of Gorr et al. [28], can only be explained by a wrong self-regulation of the feed demand by the sow and, therefore, a higher wastage of feed in traditional systems. The estimation of feed costs, based on the current price of lactation feed on the Spanish market (0.302 EUR/kg), shows that the average daily and total feed costs were similar between the ESF (EUR 2.14 and 51.58, respectively) and CON (EUR 2.17 and 52.37, respectively) groups. The estimation for the cost of producing 1 kg of weaned piglet was EUR 0.79 in Group ESF and EUR 0.88 in Group CON, which with weaning weights of 7.39 and 7.11 kg means EUR 5.8 and 6.3 per weaned piglet, respectively. The difference of EUR 0.5 per weaned piglet, in the case of large farms like the one used in this study, producing over 100,000 piglets per year, means there is a financial benefit of EUR 50,000 per year. Our results support previous studies [13,28] that, comparing traditional management and ad libitum systems (like our Group ESF), showed a higher consumption in the traditional system concomitant to a higher [13] or lower [28] weaning weight of the piglets.

Despite a lower feed consumption (or wastage) and a higher weaning weight of their litters, the sows in Group ESF showed a similar or even better (*p* = 0.06) bodyweight and back-fat depth at weaning than sows in Group CON. These results confirm better self-regulation of the feed intake with ESF systems, avoiding wastage of feed as in the case of traditional feeders. A possible explanation may be related to the fact that ESF sows have feed availability more times per day but in a lower amount, which diminishes refusal and wastage of food and allows better digestibility of diets, as described by Nielsen et al. [27]. These details may be of importance for the lifetime production of the sows because a better body condition at weaning is related to better weaning-to-estrus intervals, fertility, prolificacy and longevity [29,30,31]. On the other hand, there were no significant differences in blood metabolism indexes between sows in Groups ESF and CON; this is an expected finding since there were no sows affected by any pathology and the sows received the same diet. Similar results were found in piglets, excepting lower—but within physiological values [32]—plasma concentrations of HDL cholesterol and proteins in Group ESF at 14 days of lactation. These specific differences in the values of proteins and lipids may be related to the differences in the content of protein and fat of the milk at that moment, since they are primarily affected by the nutritional value of the feed [32,33]. Afterwards, at weaning, the metabolic parameters were again similar between groups, which may be related to the assignment of the same creep feeding to all the piglets from 14 days onwards; hence, the nutritional value of the feed is more similar between groups and creep feeding drives this value.

Overall, our results indicate an important economic impact of ESF systems when compared to traditional systems: firstly, directly, with heavier litters and sows in better body condition with lower feed intake, which means a financial benefit of EUR 0.5 per weaned piglet; secondly, indirectly, because of the developmental trajectory of the piglets produced. It is well known that producing heavier piglets and litters at weaning has provided benefits in the subsequent phases because large piglets have a higher feed intake and growth competence than smaller piglets during the nursery and growing phases [34]. Furthermore, heavier piglets require simpler diets in nutritional terms (lower content of milk powder, cooked/extruded cereals, lower percentage of soy concentrates and lower proportion of available amino acids), so the costs associated with early-age feeding, involving complex components and high protein and amino acid levels during longer periods, decrease [35,36]. Moreover, smaller weaned piglets have higher mortality during the nursery and growing periods [35], with differences reaching even 54% when compared to larger piglets [37]. Finally, heavier piglets not only have higher ADWG during the nursery and growing phases but also have fewer days-to-slaughterhouse and better carcass quality [34].

## 5. Conclusions

The results of this study indicate that, under farm conditions, the use of precision livestock farming systems and, specifically, electronic sow feeders offers remarkable technical and economic outputs compared with traditional feeders due to the weaning of heavier piglets with a lower amount of feed per kg of weaned piglet, which means a financial benefit of EUR 0.5 per weaned piglet. These findings show the importance of sows’ intake and pave the way for future research.

## Figures and Tables

**Table 1:** Trial 1. Number of piglets in each litter and weight of individual piglets and litters (range and total number are shown within parentheses) which were born from sows fed with electronic sow feeders (ESFs; 178 sows) and traditional feeders (CON; 190 sows) during the lactation period.

Group	Total Piglets	Live Piglets	Stillbirths	Litter Weight (kg)	Piglet Weight (kg)
**ESF**	17.39 ± 0.42 (5–27)	15.19 ± 0.39 (4–23)	2.26 ± 0.27 (0–17)	21.41 ± 0.33 (11.68–34.10)	1.59 ± 0.02 (1.00–2.62)
**CON**	17.14 ± 0.42 (3–30)	15.36 ± 0.39 (3–27)	1.79 ± 0.27 (0–15)	21.78 ± 0.36 (6.30–42.6)	1.61 ± 0.03 (0.87–3.06)
***p*-Value**	0.686	0.754	0.214	0.456	0.575

**Table 2:** Trial 1: Number and total weight of piglets in the litter and weight and Average Daily Weight Gain (ADWG) of individual piglets weaned from sows fed with electronic sow feeders (ESFs) and traditional feeders (CON) during the lactation period.

Group	Weaned Piglets (n)	Litter Weight (kg)	Piglet Weight (kg)	ADWG (kg/day)
**ESF**	12.02 ± 0.13	88.78 ± 1.18	7.39 ± 0.07	0.220 ± 2.86
**CON**	11.95 ± 0.14	84.78 ± 1.26	7.11 ± 0.08	0.212 ± 3.06
***p*-Value**	0.738	0.030	0.007	0.040

**Table 3:** Trial 1: Average daily and total feed intake in sows fed with electronic sow feeders (ESFs) and traditional feeders (CON) during a lactation period of 24 days in length.

Group	Average Daily Feed Intake (kg)	Total Feed Intake (kg)	Kg Feed per Kg of Weaned Piglet
**ESF**	7.08 ± 0.05	170.79 ± 1.49	2.63 ± 0.06
**CON**	7.19 ± 0.06	173.40 ± 1.58	2.93 ± 0.07
***p*-Value**	0.148	0.223	0.002

**Table 4:** Trial 2: Number of piglets in each litter and weight of individual piglets and litters which were born from sows fed with electronic sow feeders (ESFs) and traditional feeders (CON) during the lactation period.

Group	Total Piglets	Live Piglets	Stillbirths	Litter Weight (kg)	Piglet Weight (kg)
**ESF**	19.83 ± 2.71 (12–30)	16.33 ± 2.33 (8–24)	3.50 ± 1.59 (0–10)	19.99 ± 2.43 (12.04–27.33)	1.20 ± 0.03 (0.81–1.75)
**CON**	21.17 ± 1.71 (15–27)	17.83 ± 1.99 (11–24)	3.33 ± 1.38 (1–10)	20.54 ± 2.06 (12.66–25.93)	1.16 ± 0.02 (1.01–1.66)
***p*-Value**	0.681	0.635	0.938	0.868	0.304

**Table 5:** Trial 2: Bodyweight (kg) back-fat depth (mm) and Average Daily Weight Gain (ADWG) in sows fed with electronic sow feeders (ESFs) and traditional feeders (CON) during a lactation period of 24 days in length.

Group	Prior to Delivery		Weaning		ADWG
	Bodyweight	Back-Fat Depth	Bodyweight	Back-Fat Depth	
**ESF**	280.17 ± 3.92	10.57 ± 1.04	248.00 ± 4.21	8.30 ± 1.03	−1.42 ± 0.14
**CON**	274.33 ± 6.40	10.43 ± 1.14	236.50 ± 3.43	7.72 ± 0.74	−1.68 ± 0.30
***p*-Value**	0.455	0.933	0.060	0.656	0.235

**Table 6:** Trial 2: Milk components (%) in sows fed with electronic sow feeders (ESFs) and traditional feeders (CON) during lactation.

Group	Dry Matter	Protein	Fat	Lactose
**ESF**	18.69 ± 0.47	4.77 ± 0.05	6.48 ± 0.16	5.73 ± 0.08
**CON**	19.26 ± 0.34	4.96 ± 0.21	6.74 ± 0.28	5.60 ± 0.11
***p*-Value**	0.444	0.395	0.369	0.190

**Table 7:** Trial 2: Plasma concentrations for parameters of glucose, lipids and protein metabolism at weaning in sows fed with electronic sow feeders (ESFs) and traditional feeders (CON) during lactation.

Group	Glucose (mg/dL)	Fructosamine (µmol/L)	Cholesterol (mg/dL)	HDL-c (mg/dL)	LDL-c (mg/dL)	Triglycerides (mg/dL)	Proteins (g/dL)
**ESF**	90.27 ± 7.22	380.98 ± 39.89	110.13 ± 9.06	38.82 ± 7.52	42.88 ± 5.83	58.95 ± 6.23	7.66 ±0.38
**CON**	86.66 ± 7.64	412.67 ± 31.85	103.48 ± 10.54	39.09 ± 5.28	42.47 ± 4.03	59.70 ± 3.63	7.91 ± 0.20
***p*-Value**	0.739	0.549	0.754	0.403	0.206	0.162	0.577

**Table 8:** Trial 2: Plasma concentrations for parameters of glucose, lipids and protein metabolism in piglets from sows fed with electronic sow feeders (ESFs) and traditional feeders (CON) during lactation.

Group	Glucose (mg/dL)		Fructosamine (µmol/L)	Cholesterol (mg/dL)	HDL-c (mg/dL)	LDL-c (mg/dL)	Triglycerides (mg/dL)	Proteins (g/dL)
**ESF**	14	144.62 ± 3.97	367.38 ± 4.12	144.82 ± 6.05	70.25 ± 2.41	56.12 ± 4.65	124.11 ± 5.35	5.94 ± 0.11
	28	142.75 ± 3.16	366.88 ± 3.20	122.69 ± 4.37	52.60 ± 2.52	49.75 ± 1.86	112.03 ± 6.39	5.36 ± 0.09
**CON**	14	137.38 ± 6.33	372.56 ± 6.74	157.27 ± 7.11	78.47 ± 2.34	51.82 ± 2.80	135.79 ± 6.57	6.36 ± 0.13
	28	140.81 ± 3.83	375.16 ± 5.72	125.13 ± 5.17	51.67 ± 2.24	46.31 ± 2.14	122.44 ± 6.75	5.34 ± 0.08
***p*-Value**	14	0.339	0.517	0.186	0.017	0.428	0.173	0.018
	28	0.706	0.241	0.728	0.783	0.241	0.275	0.907

**Table 9:** Trial 2: Number and total weight of piglets in the litter and weight and Average Daily Weight Gain (ADWG) of individual piglets weaned from sows fed with electronic sow feeders (ESFs) and traditional feeders (CON) during the lactation period.

Group	Weaned Piglets (n)	Litter Weight (kg)	Piglet Weight (kg)	ADWG (kg/day)
**ESF**	13.00 ± 0.82	83.48 ± 5.88	6.17 ± 0.03	0.210 ± 0.01
**CON**	12.83 ± 0.70	70.14 ± 3.98	5.35 ± 0.02	0.179 ± 0.01
***p*-Value**	0.880	0.090	0.0006	0.0008

## Data Availability

All data are included in the manuscript and Supplementary Materials.

## Supplementary Materials

The following is available online at https://www.mdpi.com/article/10.3390/ani14192863/s1: Table S1: Composition and calculated analysis of the diets used in both trials.

## References

[1] Whittemore C.T., Kyriazakis I. (2006). Whittemore’s Science and Practice of Pig Production.

[2] Theil P.K., Krogh U., Bruun T.-S., Feyera T. (2023). Feeding the Modern Sow to Sustain High Productivity. Mol. Reprod. Dev..

[3] Quiniou N., Dagorn J., Gaudré D. (2002). Variation of Piglets’ Birth Weight and Consequences on Subsequent Performance. Livest. Prod. Sci..

[4] Gondret F., Lefaucheur L., Louveau L., Lebret B., Pichodo X., Le Cozler Y. (2005). Influence of Piglet Birth Weight on Postnatal Growth Performance, Tissue Lipogenic Capacity and Muscle Histological Traits at Market Weight. Livest. Prod. Sci..

[5] Beaulieu A.D., Aalhus J.L., Williams N.H., Patience J.F. (2010). Impact of Piglet Birth Weight, Birth Order, and Litter Size on Subsequent Growth Performance, Carcass Quality, Muscle Composition, and Eating Quality of Pork. J. Anim. Sci..

[6] Kansas State University Swine Nutrition Guide. General Nutrition Principles. Economics in Swine Nutrition..

[7] Sulabo R., Jacela J., Wiedemann E., Tokach M., Nelssen J., DeRouchey J., Goodband R., Dritz S. (2014). Effects of Lactation Feed Intake and Creep Feeding on Sow and Piglet Performance. Kansas Agric. Exp. Stn. Res. Rep..

[8] Hawe S.-J., Scollan N., Gordon A., Magowan E. (2020). Impact of Sow Lactation Feed Intake on the Growth and Suckling Behavior of Low and Average Birthweight Pigs to 10 Weeks of Age. Transl. Anim. Sci..

[9] Berckmans D. (2006). Automatic On-Line Monitoring of Animals by Precision Livestock Farming. Livest. Prod..

[10] Guarino M., Norton T., Berckmans D., Vranken E., Berckmans D. (2017). A Blueprint for Developing and Applying Precision Livestock Farming Tools: A Key Output of the EU-PLF Project. Anim. Front..

[11] Gómez Y., Stygar A.-H., Boumans I.-J.-M.-M., Bokkers E.-A.-M., Pedersen L.-J., Niemi J.-K., Pastell M., Manteca X., Llonch P. (2021). A Systematic Review on Validated Precision Livestock Farming Technologies for Pig Production and Its Potential to Assess Animal Welfare. Front. Vet. Sci..

[12] Mosnier E., Etienne M., Ramaekers P., Père M.C. (2010). The Metabolic Status during the Peri Partum Period Affects the Voluntary Feed Intake and the Metabolism of the Lactating Multiparous Sow. Livest. Sci..

[13] Hansen A.V., Bach K.E., Knudsen N.J., Kjeldsen H.D., Jensen B.B. (2012). Feed intake in Reproducing Sows. Nutritional Physiology of Pigs.

[14] Quiniou N. (2021). Results of 15 Years of Precision Feeding of Hyper-Prolific Gestating Sows. Animals.

[15] Gauthier R., Largouët C., Bussières D., Martineau J.P., Dourmad J.Y. (2022). Precision Feeding of Lactating Sows: Implementation and Evaluation of a Decision Support System in Farm Conditions. J. Anim. Sci..

[16] Pomar C., Hauschild L., Zhang G.H., Pomar J., Lovatto P.A. (2009). Applying Precision Feeding Techniques in Growing-Finishing Pig Operations. Rev. Bras. Zootecn..

[17] Gaillard C., Brossard L., Dourmad J.Y. (2020). Improvement of Feed and Nutrient Efficiency in Pig Production Through Precision Feeding. Anim. Feed Sci. Technol..

[18] Koketsu Y., Dial G.D., Pettigrew J.E., King V.L. (1996). The Influence of Nutrient Intake on Biological Measures of Breeding Herd Productivity. J. Swine Health Prod..

[19] Eissen J.J., Kanis E., Kemp B. (2000). Sow factors affecting voluntary feed intake during lactation. Livest. Prod. Sci..

[20] Solà-Oriol D., Gasa J. (2017). Feeding Strategies in Pig Production: Sows and their Piglets. Anim. Feed Sci. Technol..

[21] De Blas C., Gasa J., Mateos G.G. (2013). Necesidades Nutricionales para Ganado Porcino. Fundación Española para el Desarrollo de la Nutrición Animal.

[22] Klindt J. (2003). Influence of Litter Size and Creep Feeding on Pre-Weaning Gain and Influence of Preweaning Growth on Growth to Slaughter in Barrows. J. Anim. Sci..

[23] Hartmann P.E., Smith N.A., Thompson M.J., Wakeford C.M., Arthur P.G. (1997). The Lactation Cycle in the Sow: Physiological and Management Contradictions. Livest. Prod. Sci..

[24] Sadovnikova A., Garcia S.-C., Hovey R.-C. (2021). A Comparative Review of the Cell Biology, Biochemistry, and Genetics of Lactose Synthesis. J. Mammary Gland. Biol. Neoplasia.

[25] Gaines W.L. (1940). Milk Energy Yield and the Correlation between Fat Percentage and Milk Yield. J. Dairy Sci..

[26] Krogh U. (2017). Mammary Plasma Flow, Mammary Nutrient Uptake and the Production of Colostrum and Milk in High-Prolific Sows: Impact of Dietary Arginine, Fiber and Fat. Ph.D. Thesis.

[27] Nielsen S.-E., Bache J.-K., Bruun T.-S., Strathe A. Effect of Feeding Strategy on Piglet Growth and Survival and Milk Production of Loose-Housed Lactating Sows. https://ssrn.com/abstract=4671037.

[28] Gorr S.-C., Leeb C., Zollitsch W., Winckler C., Parsons T.D. (2024). Ad libitum Feeding Systems for Lactating Sows: Effects on Productivity and Welfare of Sows and Piglets. Animal.

[29] Dourmad J.Y., Etienne M., Prunier A., Noblet J. (1994). The Effect of Energy and Protein Intake of Sows on their Longevity: A Review. Livest. Prod. Sci..

[30] Johnston L.J., Fogwell R.N., Weldon W.C., Ames N.K., Ullrey D.E., Miller E.R. (1989). Relationship between Body Fat and Postweaning Interval to Estrus in Primiparous Sows. J. Anim. Sci..

[31] Yoder C.L., Schwab C.R., Fix J.S., Stalder K.J., Dixon P.M., Duttlingere V.M., Baas T.J. (2013). Estimation of Deviations from Predicted Lactation Feed Intake and the Effect on Reproductive Performance. Livest. Sci..

[32] Petrovic V., Novotný J., Hisira V., Link R., Leng L., Kovac G. (2009). The Impact of Suckling and Post-weaning Period on Blood Chemistry of Piglets. Acta Vet. Brno.

[33] Lauridsen C., Jensen S.K. (2007). Lipid Composition of Lactational Diets Influences the Fatty Acid Profile of the Progeny before and after Suckling. Animal.

[34] Moest N.K., Willard N.C., Shull C.M., McKilligan D., Ellis M. (2023). 187 Effect of Piglet Weaning Weight on Wean-to-Finish Growth Performance and Ultrasound Carcass Measures. J. Anim. Sci..

[35] Pomar C., Remus A. (2019). Precision Pig Feeding: A Breakthrough toward Sustainability. Anim. Front..

[36] Collins C.L., Pluske J.R., Morrison R.S., McDonald T.N., Smits R.J., Henman D.J., Stensland I., Dunshea F.R. (2017). Post-Weaning and Whole-of-Life Performance of Pigs is Determined by Live Weight at Weaning and the Complexity of the Diet Fed after Weaning. Anim. Nutr..

[37] Huting A.M.S., Wellock I., Tuer S., Kyriazakis I. (2019). Weaning Age and Post-Weaning Nursery Feeding Regime are Important in Improving the Performance of Lightweight Pigs. J. Anim. Sci..

